# Importance of In Vitro Embryo Model Procedure Standardization

**DOI:** 10.1002/jcla.25082

**Published:** 2024-07-29

**Authors:** Kubilay Doğan Kılıç, Zeynep Simge Yılmaz

**Affiliations:** ^1^ Department of Histology and Embryology, Faculty of Medicine Ege University İzmir Türkiye; ^2^ Institute for Tissue Engineering and Regenerative Medicine Helmholtz Zentrum München Germany

**Keywords:** embryo studies, in vitro studies, in vivo studies, standardization, standardized protocols

## Abstract

In vivo studies offer a detailed understanding of organism functioning, surpassing the insights provided by in vitro studies. These experiments are crucial for comprehending disease emergence, progression, and associated mechanisms in humans, as well as for developing treatments. When choosing experimental models, factors such as genomic similarity, physiological relevance, ethical appropriateness, and economic feasibility must be considered. Standardized protocols enhance the reliability, and reproducibility of scientific methods, promoting the assessment of research in the scientific literature. Researchers conducting embryo studies should establish and document standardized protocols for increased data comparability. Standardization is vital for scientific validity, reproducibility, and comparability in both in vivo and in vitro studies, ensuring the accuracy and reliability of experimental results and advancing scientific knowledge.

In vivo studies allow us to obtain more detailed information about the functioning of organism than in vitro studies. In vivo experimental models are frequently used to understand the cause of the emergence of diseases in humans, the progression of the disease, and the mechanisms that affect this process and to develop treatments for various diseases. When creating experimental models, the similarity of the genome of the species to be used to the human genome, its suitability for human physiology and anatomy, the easy availability of the species, ethical suitability for use and economic accessibility are the issues that should be taken into consideration [1].

There are experimental models and protocols that have been widely used to reproduce various diseases in in vivo studies from the past to the present.

Streptozotocin is often used in diabetes modeling. In Parkinson's models, rotenone is often used [2]. In addition, knock‐out and knock‐in mouse models are often used to understand the functioning of a gene and the mechanisms it affects [3].

In vivo studies are again of great importance in cancer modeling and treatment. Rats are useful models for the study of induced breast cancer. Their ability to develop mammary tumors with histopathology comparable to humans has made them frequently preferred animals for cancer modeling [4].

All these examples, which are not directly related to each other, lead us to a single point. Standardized models and methods allow us to obtain more detailed, practical, and repeatable data on the subject we want to study.

In vivo studies have made indisputable contributions to humanity by illuminating the unknowns for diseases and their treatments from past to present and continue to guide scientists in this field. While there are important models for post‐implantation development of the embryo, many of these models lack information on extra‐embryonic lineages and cell types [5]. Given these reasons, in vivo embryo studies have some flaws and drawbacks.

The embryonic period between embryonic days 0 and 20 is a black box for researchers, as there are technical and ethical barriers to frequent sample collection to study the embryo in utero. After fertilization, the human embryo is a collection of stem cells that begin to build the life of the organism and undergo morphogenetic transformations once implanted in the uterus. Knowledge of this crucial stage remains incomplete due to lack of ability to observe the embryo in vivo [6]. Even if it is possible to create an ex vivo culture with structures obtained from human blastocysts, the reproducibility of the structural organization that occurs in in vivo embryos is in most cases coincidental [7].

Embryo studies face many legal and ethical challenges. Researchers need to overcome ethical and technical challenges in order to learn about the less understood early stages in the formation of human life.

Researchers may encounter problems with natural mating in in vivo studies. During the research, problems with the timing of fertilization may occur, and fertilization success is also under the influence of hormonal and environmental factors. If researchers do not know the mating periods of living organisms clearly in in vivo experiments and fertilization does not occur during estrus, the study may be interrupted because of the loss of time in the research. For example, even the vaginal plaque, which is often used in embryonic toxicology studies, does not have 100% success and is unsafe for studies where days and hours are more important than term [8]. Although we can overcome this problem with artificial insemination, this technique is also work‐dependent and uneconomical as well as not ergonomic for every laboratory [9].

The general definition in the current literature for embryo models is in vitro, as the technique is established, but in terms of application, it is similar to in vivo studies. As studies become more frequent, it is likely that a new term will be needed to describe both the in vitro methodology and the in vivo nature of this intermediate stage. We propose the term *in speculo* to encompass both the in vitro materials of these models and the use of the results as a mirror of life. While in vitro studies alone cannot replace in vivo studies, embryo models, free from ethical problems and the influence of environmental conditions, can take an important place between these two fields of study.

When we examined the current situation in reputable literature, we wanted to start by quoting Sozen et al.'s work as they are also a citizen of the source country of this editorial. Sozen et al.'s work on “Artificial Embryos,” in which they were the lead researcher, was included in the Massachusetts Institute of Technology's “10 Breakthroughs of the Year” list in 2018 [10]. In this innovative study, Sozen et al. aimed to test whether cross‐talk between the three stem cell types present in the natural embryo could enable the generation of whole embryo‐like structures capable of exhibiting the cellular rearrangements of gastrulation. For this purpose, they created a culture circumstance for the stem cell lines of the three tissues that make up the mouse embryo which is embryonic stem cells, trophoblast stem cells, and extra‐embryonic endoderm cells to cooperate in vitro. They showed that this cooperation results in the spontaneous formation of structures that closely resemble natural mouse embryos, including all three embryonic and extra‐embryonic compartments [11].

In their review, Sozen et al. emphasized that due to the need for a human‐centered understanding of embryogenesis, novel in vitro approaches that drive human pluripotent stem cells to form embryonic organoids that model embryo development are crucial. They described these new technologies outlining aspects of human development in his work. They showed how these technologies can provide insights into the molecular, cellular, and morphogenetic processes that fuel the formation of a full fetus, highlighting the potential of these platforms to revolutionize the understanding of human development in health and disease. The creation of in vitro models of mammalian embryo development from pluripotent stem cells is referred to as “stembryos” in this study, but the terms embryonic organoids, embryoids, and stem cell‐based embryo models are also used. These model systems are said to be able to recreate the complex features of human embryonic development in vitro. In this study, they provide an overview of models of the developing embryo, together with recent insights from mouse and human embryos. In particular, they show that state, form, and function that are tightly coupled in vivo can be disconnected in vitro, and that such a disconnection should be taken into account when comparing models of embryo development. The researchers noted that a thorough evaluation of existing models, a better understanding of in vivo design principles, and incorporation of knowledge from mouse in vitro systems are all considerations in building advanced models of human embryogenesis [12].

A model of the post‐implantation human embryo or human embryoid made up of extraembryonic and embryonic tissues, was developed by Weatherbee et al. Through the combination of wild‐type embryonic stem cells with two types of extraembryonic‐like cells developed through transcription factor overexpression, scientists were able to drive their self‐organization into structures that resemble different parts of post‐implantation human embryo. These self‐organizing aggregates are composed of extraembryonic‐like tissues encircling a pluripotent epiblast‐like region. According to functional investigations conducted by Weatherbee and colleagues, in response to stimuli involving bone morphogenetic proteins, the epiblast‐like domain develops considerably into amnion, extraembryonic mesenchyme, and primordial germ cell‐like cells. In addition, they identified an inhibitory role for SOX17 in the specification of anterior hypoblast‐like cells. In this study, they established a multi‐stage stem cell‐derived model of the human post‐implantation embryo. Their stem cell‐derived inducible human embryo model generates amnion‐like cells that mature progressively in response to BMP signaling. Primordial germ cell‐like cells readily differentiate in the established human embryo stem cell model and these cells are identified along the amnion differentiation trajectory. With this study, they provide evidence that they originate from a common AP2α‐positive progenitor as reported in other in vitro systems [6].

In their review, Moris emphasized the use of stem cells to create models that bypass the early stages of development and resemble a 2‐week‐old human embryo, and that this was a turning point for studies that were not possible in human embryos. Once the appropriate environment and conditions are created for the growth of these constructs, they allow pure human stem cells to spontaneously differentiate into other cell types under specific chemical conditions [13].

Oldak et al. noted that the study of post‐implantation development in humans is limited due to ethical and technical challenges. Implantation of the human embryo leads to a number of changes and much of this process is based on the morphogenesis of extra‐embryonic tissues and its effect on the organization of embryonic cells, the researchers said, noting that this is a stage with a high incidence of pregnancy loss, they emphasized that understanding events related to this developmental period would be of great benefit in understanding developmental defects associated with fertility, but noted that there are justifiable ethical obstacles to conducting such studies with material derived from donated human embryos. The importance of creating complete embryo models with human stem cell‐derived models that can mimic conditions that play an important role in early post‐implantation developmental processes is thus once again recognized. Researchers have developed a protocol in mice to generate self‐organizing post‐implantation SEMs from pure embryonic stem cells without the need to establish cell lines with embryo tissue samples. In this way, they have shown that only the generation of pure pluripotent stem cells can be sufficient to produce embryo‐like structures that develop outside the uterus in mammals, including humans [7].

Pedroza et al. used human pluripotent stem cells sustained under conditions supporting a state of intermediate pluripotency, and these cells were harvested in 3D and exposed to spontaneous differentiation media created for this study, which provided minimal growth factor support. Within 48 h, they observed that these human pluripotent stem cells self‐organized into various cell types. With this study, the researchers report a novel in vitro approach to efficiently self‐assemble to recapitulate key features of human perigastrulation [5].

Whether they are called stembryos, embryoids or by more traditional names, these studies, which differ from each other in significant unique ways, have been finalized by adding high technology to their accessible infrastructure as stones on the way to the same goal. If these distinguished studies, which use a large number of high‐level methods or diversity within the same method (e.g., antibodies), are to achieve reproducibility and widespread use, they need to move toward an ideal reduced methodology in the future. In this case, developmental studies, teratonije studies and toxicology studies will enter a new era.

In parallel with all these new generation studies, our thoughts are as follows: the ethical and legal framework of in vivo and in vitro studies should be meticulously evaluated. In sensitive issues such as human embryo studies, full compliance with international standards and local legal regulations should be ensured.

One of the important points about standardization and protocols is the establishment of universal experimental protocols; we see groundbreaking materials and methods in every new and original study. Nevertheless, the commonalities of these studies can form the basis for common protocols. If any changes to the protocols need to be made during the research, these changes should be documented and explained. This ensures that future researchers can repeat the experiments.

The laboratory environment for in vitro studies needs to be constantly monitored and controlled in terms of temperature, humidity, and other factors. This helps to minimize external factors that may affect the results of the experiment.

Standardization and protocols increase the reliability of scientific methods and ensure that scientific results can be reproduced by other researchers. This makes it possible to evaluate research in a way that contributes to the scientific literature. Researchers should establish standardized protocols for embryo studies and document these protocols in detail. This increases the comparability of data. Standardization and protocols are essential for the scientific validity, reproducibility, and comparability of research processes. Standardization of in vivo and in vitro studies improves data quality in the scientific community and ensures the accuracy and reliability of experimental results.

## Conflicts of Interest

The authors declare no conflicts of interest.

## Data Availability

Data sharing not applicable to this article as no datasets were generated or analysed during the current study.
